# Supplementary material for: Surveillance on California dairy farms reveals multiple possible sources of H5N1 influenza virus transmission
Source: PLoS Biol. 2026 May 5;24(5):e3003761. doi: 10.1371/journal.pbio.3003761 (PMC13143106; doi:10.1371/journal.pbio.3003761)
Supplement: S1 Table — (PDF) [file pbio.3003761.s004.pdf]

S1 Table. Details of 14 dairy farms that were sampled in California.

Dairy Farm	Location (region)	Date of H5 positivity	Approx. Number of Cows in Milk[a]	Primary Housing Pen Style	Milking Parlor Style
**BB**	Central Valley	11/23/24	800	Freestall	Parallel
**BC**	Central Valley	10/24/24	2900	Freestall	Rotary and Single Flat
**BD**	Central Valley	11/25/24	1000	Freestall	Parallel
**BF**	Central Valley	11/26/24	3000	Freestall	Rotary
**BM**	Central Valley	11/25/24	1400	Freestall	Herringbone
**EA**	Southern CA	3/3/25	300	Open Lot	Herringbone
**EB**	Southern CA	2/4/25	970	Open Lot	Unknown
**EC**	Southern CA	2/24/25	420	Open Lot	Herringbone
**ED**	Southern CA	12/2024*	500	Open Lot	Unknown
**EE**	Southern CA	12/2024*	1700	Open Lot	Herringbone
**EF**	Southern CA	12/2024*	1200	Open Lot	Herringbone
**EG**	Southern CA	2/26/24	500	Open Lot	Herringbone
**FA**	Central Valley	11/11/24	2,000	Freestall	Herringbone
**FB**	Central Valley	11/19/24	3,342	Freestall	Rotary

* Approximate date provided

a - Cows in milk refers to the number of cows currently lactating and being milked on a dairy farm, not the total number of animals on a farm.
