# Supplementary material for: Surveillance on California dairy farms reveals multiple possible sources of H5N1 influenza virus transmission
Source: PLoS Biol. 2026 May 5;24(5):e3003761. doi: 10.1371/journal.pbio.3003761 (PMC13143106; doi:10.1371/journal.pbio.3003761)
Supplement: S2 Table — (PDF) [file pbio.3003761.s005.pdf]

S2 Table. Details of air sampling during the initial phase on farms in the Central Valley of California in late 2024.

Air Sampler	Modification	Sample Location	Sample Descriptor	Farm	Date	Days post BTM+[a]	Positive/ Total
**AirPrep Cub 210 (200 LPM)**	**None**	Milking Parlor	Cows from multiple pens during milking. Sample duration 60 minutes	BC	10/29/24	5	0/4
				BC	10/30/24	6	0/3
				BC	11/4/24	11	0/2
				BC	11/7/24	14	0/1
				BC	11/11/24	18	0/2
				BM	12/4/24	9	0/2
		Housing Area[b]	Open-air pen of hospital cows[c] Sample duration 60 minutes	BM	12/4/24	9	0/1
**Open Face PTFE (5 LPM)**	**Backpack[d]**	Milking Parlor	Following milking process during milking of hospital pen[c] cows. Sample duration 20- 45 minutes	BC	10/29/24	5	0/3
				BC	10/30/24	6	0/3
				BC	11/4/24	11	0/1
				BB	12/16/24	23	0/1
			Following milking process during milking of cows recovered from clinical H5 signs	BD	12/18/24	23	0/1
			Following the parlor worker closely during milking of hospital pens of cows	BM	12/19/24	24	**1/1**
		Housing Area	Sampling of background air in housing pens. Sample duration 20- 45 minutes	BC	10/29/24	5	0/1
				BB	12/16/24	23	0/1
				BF	12/18/24	22	0/1
	**Cone[e]**	Milking Parlor	Cone directed at milking process, following worker during milking of healthy cows	BB	12/16/24	23	0/1
			Cone directed at milking process, following worker during milking of cows recovered from H5 clinical signs	BD	12/18/24	23	0/1
		Housing Area	Exhaled breath of 15 - 30 cows in the hospital pen; cone held close to muzzles. Sample duration 5- 15 seconds/cow	BM	12/16/24	21	0/1
				BF	12/18/24	22	0/1
				BM	12/19/24	24	0/2
			Exhaled breath of individual cow with 105F fever on 12/16/25 in hospital pen. Sample duration 2 minutes	BM	12/16/24	21	0/1
				BM	12/19/24	24	0/1
	**No Cone**	Housing Area	Exhaled breath of individual hospital pen cows. Sample duration 5- 15 minutes	BC	10/29/24	5	0/6
				BC	10/30/24	6	0/8
				BC	11/4/24	11	0/2
**MD8 Airport (50 LPM)**	**Cone**	Milking Parlor	Cone directed at milking process, following worker, during milking of a healthy pen of cows. Sample duration 10 minutes	BB	12/16/24	23	0/2
			Cone directed at milking process, following worker, during milking of the hospital pen. Sample duration 10 minutes	BB	12/16/24	23	0/2
			Cone directed at milking process, following worker during milking of cows recovered from H5 clinical signs. Sample duration 10 minutes	BD	12/18/24	23	0/1
			Following the milking process for hospital pens of cows; closely following parlor worker throughout. Sample duration 5- 15 minutes	BM	12/19/24	24	**3/3**
		Housing Area	Exhaled breath of 15-30 cows, cone held close to muzzles. Sample duration 5- 15 seconds/cow	BB	12/16/24	23	0/1
			Exhaled breath of 15 - 30 clinically recovered cows; cone held close to muzzles. Sample duration 5- 15 seconds/cow	BD	12/18/24	23	0/1
			Exhaled breath of 15-30 hospital pen cows; cone held close to muzzles. Sample duration 5- 15 seconds/cow	BF	12/18/24	22	0/1
				BM	12/17/24	22	0/2
				BM	12/19/24	24	**2/2**
			Exhaled breath of individual cow in hospital pen that had an H5+ nasal swab on 12/4/24. Sample duration 1- 2 minutes	BM	12/16/24	21	0/2
			Exhaled breath of individual cow with severe signs associated with H5, cow couldn't stand. Sample duration 2- 3 minutes	BF	12/18/24	22	0/1
			Exhaled breath of individual cow in hospital pen that had a 105F fever on 12/16/24. Sample duration 1-2 minutes	BM	12/19/24	24	0/1

a- Days post BTM+ - Days post first bulk tank milk positive

b - Housing Area refers to primary pens used to house cows; these can be open-air or freestall pens depending on individual dairy.

c - Hospital cows/pen refers to animals identified by farmers, according to internal criteria, that have clinical signs requiring their milk not go to the bulk tank.
These animals are grouped into 'hospital pens' separated from healthy animals.

d - Backpack refers to wearing the PTFE filter as shown in Fig 1D; mimicking occupational exposure

e- Cone refers to using a plastic cone adapted to the front of the air sampler as shown in Fig 1D, to prevent aerosol dilution and to protect filters from direct splashes.
