# Supplementary material for: Surveillance on California dairy farms reveals multiple possible sources of H5N1 influenza virus transmission
Source: PLoS Biol. 2026 May 5;24(5):e3003761. doi: 10.1371/journal.pbio.3003761 (PMC13143106; doi:10.1371/journal.pbio.3003761)
Supplement: S3 Table — (PDF) [file pbio.3003761.s006.pdf]

S3 Table. Air sampling details for dairies with 1 or less positive environmental samples, Feb-Apr 2025

Sample Location	Sample Type	Sample Source	Sample Descriptor	Farm	Date	Days post BTM+[a]	Positives/ Total
Milking Parlor	Milk	Bulk Tank	Collection from bulk tank	EA	2/26/25	-5	0/1
				EA	2/27/25	-4	**1/1**
				EA	3/4/25	1	**1/1**
				EE	3/2/25	~ 90	0/1
				FA	4/1/25	141	0/1
				FA	4/2/25	142	0/1
				FA	4/3/25	143	0/1
				FB	4/1/25	133	0/1
				FB	4/2/25	134	0/1
				FB	4/3/25	135	0/1
		Milk from individual cows, 4 teats	Collected from cows sorted into the hospital pen[b] for signs such as mastitis and potential *E. coli* infection	EF	3/2/25	~90	0/3
			Collected from cows with abnormal milk consistent with descriptions of milk from H5+ cows such as yellowy color and thick consistency.	FB	4/2/25	134	0/2
		Bulk Sick Cow Milk	Bulk milk from cows whose milk didn't go to bulk tank. No specific clinical signs described	FA	4/1/25	141	0/1
			Bulk milk from cows whose milk didn't go to bulk tank. Abnormal milk only clinical sign described.	FB	4/2/25	134	0/1
	Air	Milking Process	MD8 Airport: cone directed at milking process; following worker. Sample duration 5- 15 minutes	EA	2/26/25	-5	0/1
				EF	3/2/25	~90	0/1
			Open Face PTFE worn on backpack following worker during milking. Sample suration 58 minutes	EE	3/2/25	~90	0/1
	Surface Swab	Milking Unit Inflations	Swab of the interior of all 4 inflations of one milking unit	EE	3/2/25	~90	0/2
Wastewater Stream	Air	Milk Line Cleanout	MD8 Airport held at close range to where wastewater from the milk lines exits the parlor to gravity-flow to the 'flush pump pit' below the manure lagoon. Sample duration 7 minutes	FB	4/2/25	134	0/1
		Sump pump	MD8 Airport: primary sump pump sampled while water flowing. Sample duration 7 minutes	EA	2/26/25	-5	0/1
			MD8 Airport lowered into large, deep sump pump while wastewater flowing. Sample duration 6 minutes	FA	4/1/25	141	0/1
				FA	4/2/25	142	0/1
			Open Face PTFE: primary sump pump sampled while water flowing. Sample duration 12 minutes	EA	2/27/25	-4	0/1
		Flush pump pit[c]	MD8 Airport held over area of most agitation of the flush pump pit. Sample duration 5 minutes	FB	4/1/25	133	0/2
				FB	4/2/25	134	0/1
				FB	4/3/25	135	0/1
		Manure Lagoon	MD8 Airport held, or suspended on a telescoping pole, over inlet for milking parlor wastewater into the lagoon. Sample duration 5- 9 minutes	EA	2/26/25	-5	0/1
				ED	3/2/25	~90	0/1
				EE	3/2/25	~90	0/1
				EF	3/2/25	~90	0/1
				FA	4/1/25	141	0/1
		Flush of Freestall Pens[d]	MD8 Airport held right in front of where water is pumped up into the freestall pens, vigorous water flow. Sample duration 5- 8 minutes	FB	4/1/25	133	0/2
				FB	4/2/25	134	0/1
				FB	4/3/25	135	0/1
	Wastewater	Milk Line Cleanout	Sample of residual milk and wastewater flushed out of lines as part of the cleaning process post- milking	ED	3/1/25	~90	0/1
				EF	3/2/25	~90	0/1
				EE	3/2/25	~90	0/1
				FB	4/2/25	134	0/1
		Sump pump	1L from primary pump while water flowing	EA	2/26/25	-5	0/1
				EA	2/27/25	-4	0/2
				EA	3/4/25	1	0/1
			1L from secondary pump while water flowing	EA	2/26/25	-5	0/1
				EA	2/27/25	-4	0/1
				EA	3/4/25	1	0/1
			1L sample from deep pump while wastewater flowing.	FA	4/1/25	141	0/2
				FA	4/2/25	142	0/1
				FA	4/3/25	143	0/1
		Flush pump pit	1L sample of wastewater, lots of agitation.	FB	4/1/25	133	0/1
				FB	4/2/25	134	0/1
				FB	4/3/25	135	0/1
		Manure Lagoon	1L sample from pipe inlet to lagoon	EA	3/4/25	1	0/1
				ED	3/2/25	~90	0/1
				EE	3/2/25	~90	0/1
				EF	3/2/25	~90	0/1
			1L sample from lagoon milking parlor wastewater was flowing into	FA	4/1/25	141	0/3
				FA	4/2/25	142	0/1
				FA	4/2/25	143	0/1
		Field	1L sample from pump adding a 1:1 mix of wastewater from the manure lagoon to well water onto in-use crop field	FA	4/1/25	141	**1/1**
		Flush of Freestall Pens	250mL sample of water collected as it flowed out into the pens.	FB	4/1/25	133	0/1
				FB	4/2/25	134	0/1
				FB	4/3/25	135	0/1
Housing Pens	Air	Exhaled Breath of Row of Cows	MD8 Airport held very close to ~ 12 cows' muzzles as they were headlocked into stanchions. Hospital pen sampled. Sample duration 10- 30 seconds/cow	EF	3/2/25	~90	0/1

a- Days post BTM+ - Days post first bulk tank milk positive

b - Hospital cows/pen refers to animals identified by farmers, according to internal criteria, that have clinical signs requiring their milk not go to the bulk tank.

c - The flush pump pit is a large basin that was used to mix wastewater from the manure lagoon with fresh water. Water from this pit is used to clean other areas of the dairy, such as freestall pens.

d - The freestall pens are flushed daily with water to clean them out. On the sampled farm this water came from the flush pump pit.
