# Supplementary material for: Surveillance on California dairy farms reveals multiple possible sources of H5N1 influenza virus transmission
Source: PLoS Biol. 2026 May 5;24(5):e3003761. doi: 10.1371/journal.pbio.3003761 (PMC13143106; doi:10.1371/journal.pbio.3003761)
Supplement: S4 Table — (PDF) [file pbio.3003761.s007.pdf]

S4 Table. Sampling details for dairy farm EB during spring 2025.

Sample Location	Sample Type	Sample Source	Sample Descriptor	Date	Days post BTM+[a]	Positives/ Total
Wastewater Stream	Air	Sump pump	MD8 Airport (50 LPM) sampling for 7 minutes while pump cycled	2/26/25	22	0/1
				2/27/25	23	0/1
		Field	MD8 Airport (50 LPM) sampling over the wastewater outlet in the field for 4- 6 minutes	2/26/25	22	0/2
				3/1/25	25	0/1
				3/4/25	28	0/1
	Wastewater	Sump pump	1L sample from sump pump	2/26/25	22	**1/1**
				2/27/25	23	**1/1**
		Field	1L sample from wastewater inlet to field, usually while pump running	2/26/25	22	**3/3**
				3/1/25	25	**1/1**
				3/4/25	28	**1/1**
		Manure Lagoon	1L sample; milkhouse wastewater not flowing here, lagoons only had run-off from housing pens and rainwater	2/26/25	22	**1/2**

a- Days post BTM+ - Days post first bulk tank milk positive
