# Supplementary material for: Surveillance on California dairy farms reveals multiple possible sources of H5N1 influenza virus transmission
Source: PLoS Biol. 2026 May 5;24(5):e3003761. doi: 10.1371/journal.pbio.3003761 (PMC13143106; doi:10.1371/journal.pbio.3003761)
Supplement: S5 Table — (PDF) [file pbio.3003761.s008.pdf]

S5 Table. Sampling details for dairy farm EC during spring 2025.

Sample Location	Sample Type	Sample Source	Sample Descriptor	Date	Days post BTM+[a]	Positives/ Total
Milking Parlor	Milk	Bulk Tank	Collection from bulk tank	2/27/25	3	**1/1**
				2/28/25	4	**1/1**
				3/1/25	5	**1/1**
				3/6/25	10	**1/1**
				3/7/25	11	**1/1**
				3/8/25	12	**1/1**
				3/9/25	13	**1/1**
				LS: 3/18 - 3/27[b]	22- 31	**10/10**
		Sick cow milk - 4 teats combined	Collected from cows with signs such as mastitis or a sudden stop in milk production.	3/1/25	5	**1/1**
				3/4/25	8	**1/2**
				3/6/25	10	**5/5**
				3/7/25	11	**5/6**
				3/9/25	13	**2/2**
		Dump bucket	Collection from communal bucket used for cows whose milk was discarded.	3/7/25	11	**1/1**
	Air	Milking process, following worker	MD8 Airport (50 LPM) with cone directed at milking process, closely following worker. Sample duration 3- 15 minutes.	2/28/25	4	**1/4**
				3/1/25	5	**1/1**
				3/4/25	8	**1/1**
				3/6/25	10	**4/5**
				3/7/25	11	**4/4**
				3/8/25	12	**4/4**
				3/9/25	13	**3/4**
				LS: 3/18 - 3/27[b]	22- 31	**5/14**
		Milking process, following worker	Open face PTFE (5 LPM) worn on backpack during parlor sampling while milking going on. Sampling duration from 3 hours 11 minutes to 4 hours 47 minutes.	2/28/25	4	0/1
				3/6/25	10	**1/1**
				LS: 3/18 - 3/27[b]	22- 31	0/4
	Surface Swab	Milking unit Inflations	Swab of the interior of all 4 inflations of one milking unit.	2/28/25	4	**1/4**
				3/1/25	5	**1/1**
				3/4/25	8	**1/1**
				3/6/25	10	**2/2**
				3/7/25	11	**1/1**
				3/8/25	12	**2/2**
				3/9/25	13	**1/1**
		Milking unit shells	Swab of the exterior of all 4 shells, or teatcups, of a milking unit.	3/1/25	5	**1/1**
				3/6/25	10	**1/1**
				3/7/25	11	**1/1**
				3/8/25	12	**1/2**
				3/9/25	13	0/1
Wastewater Stream	Air	Manure Lagoon	MD8 Airport (50 LPM) at close range to point of outlet for wastewater into the manure lagoon. Sampling from 3- 6 minutes.	2/27/25	3	0/1
				3/1/25	5	0/1
				3/4/25	8	0/1
				LS: 3/18 - 3/27[b]	22- 31	0/1
		Sump pump	MD8 (50 LPM) held over an open sump pump pit as wastewater from the milking parlor line cleaning flowed through. Sampling for 13 minutes.	3/4/25	8	**1/1**
	Wastewater	Milk line cleanout	Sample of bulk milk flushed out of lines as part of the cleaning process post-milking	3/4/25	8	0/1
				3/9/25	13	**1/1**[c]
				LS: 3/18 - 3/27[b]	22- 31	**7/7**
		Sump pump	1L sample of wastewater from sump pump pit	3/4/25	8	**1/1**
				LS: 3/18 - 3/27[b]	22- 31	**4/7**
		Manure Lagoon	1L sample sample from right next to wastewater inlet to lagoon	2/27/25	3	0/1
				3/1/25	5	**1/1**
				3/4/25	8	**1/1**
				LS: 3/18 - 3/27[b]	22- 31	**7/7**
Housing Pens	Air	Exhaled Breath of Row of On-Study Cows[d]	MD8 Airport (50 LPM) held very close to cows' muzzles. 10- 30 seconds per cow.	LS: 3/18 - 3/27[b]	22- 31	0/3[e]

a- Days post BTM+ - Days post first bulk tank milk positive
b - LS, Longitudinal Study. Samples collected between 3/18/25 and 3/27/25 are aggregated.
c - This sample analyzed via qRT-PCR instead of ddPCR, thus it is not shown in Figure 3B.
d - On-study cows refers to the group of 14 cows that were selected for the longevity study wherein daily milk samples from each teat were collected.
e- Not all 14 on-study cows were available for each day sampling conducted
