# Supplementary material for: Surveillance on California dairy farms reveals multiple possible sources of H5N1 influenza virus transmission
Source: PLoS Biol. 2026 May 5;24(5):e3003761. doi: 10.1371/journal.pbio.3003761 (PMC13143106; doi:10.1371/journal.pbio.3003761)
Supplement: S6 Table — (PDF) [file pbio.3003761.s009.pdf]

S6 Table. Sampling details for dairy farm EG during spring 2025.

Sample Location	Sample Type	Sample Source	Sample Descriptor	Date	Days post BTM+ [a]	Positives/ Total
Milking Parlor	Milk	Bulk Tank	Collection from bulk tank	3/6/25	8	1/1
				3/7/25	9	1/1
				3/8/25	10	1/1
				3/9/25	11	1/1
		Sick cow milk - 4 teats combined	Collected from cows sorted into the hospital pen that had mastitis at time of sampling.	3/5/25	7	1/3
		Bulk sick cow milk	Milk from sick cows that was diverted from the bulk tank into a smaller tank for a calf raising facility. Clinical signs of cows were decreased appetite and milk production, increased nasal discharge, and increased respiration rates.	3/6/25	8	2/2
	Air	Milking process, following worker	MD8 Airport (50 LPM) with cone directed at milking process, closely following worker during milking of the hospital pen cows. Sample duration was 10- 20 minutes.	3/5/25	7	0/1
				3/6/25	8	0/1
				3/7/25	9	0/1
				3/8/25	10	1/1
				3/9/25	11	1/1
Wastewater Stream	Wastewater	Field	1L sample from right next to pipe where wastewater from the parlor outlets into the field.	3/4/25	6	1/1
Housing Pens	Air	Exhaled breath of row of cows	MD8 Airport (50 LPM) held very close to cows' muzzles as they were headlocked into stanchions. Hospital pen sampled. ~15- 30 cows were sampled for 10- 30 seconds per cow.	3/7/25	9	0/2
				3/8/25	10	0/1
				3/9/25	11	2/3

a- Days post BTM+ - Days post first bulk tank milk positive
