# Supplementary material for: Surveillance on California dairy farms reveals multiple possible sources of H5N1 influenza virus transmission
Source: PLoS Biol. 2026 May 5;24(5):e3003761. doi: 10.1371/journal.pbio.3003761 (PMC13143106; doi:10.1371/journal.pbio.3003761)
Supplement: Attachment — Submitted filename: 260305_PlosBio_Response to reviewers Final.docx [file pbio.3003761.s012.docx]

**Response to reviewers**

PBIOLOGY-D-25-03450R1
Surveillance on California dairy farms reveals multiple sources of H5N1 transmission
PLOS Biology

Reviewer’s comments and questions will appear in black text, *responses will appear in italic blue text*. Any line numbers included in the responses are highlighted in the marked up version of the manuscript.

**Reviewers' Comments:**

**Reviewer #1 (Ari Machtinger and Dave O'Connor):**

The authors conducted multiple types of environmental surveillance on dairy farms in California to look for evidence of HPAI H5N1 viral RNA, live virus, and antibodies. They suggest that the evidence of what they detect in the environment reveals multiple potential sources of transmission. This evidence includes detection of H5N1 viral RNA in the air from aggregated exhaled breath samples and from the air in milking parlors, and infectious virus from the air in milking parlors and from water from farm wastewater sources. The authors also demonstrate the presence of variants that are potentially concerning for cow to human zoonosis, that cows can have H5N1 antibodies without the common symptoms of mastitis suggesting subclinical infection, and that the udders of cows do not demonstrate patterns of infection that would be consistent with transmission due to shared milking equipment. All together, they show that transmission may
occur by shared milking equipment, by shared/repurposed wastewater, and by aerosol transmission from the breath of cows or due to the milking process. We have three major comments and several minor comments that we recommend the authors consider to improve the manuscript.
*Response: We thank the reviewers for their comments and have addressed each below.*

Major comments
- The DOI links are not functional as listed for data availability and code availability. We could not tell whether this was due to embargo or a repository issue. In the event that the manuscript is published, raw and interpreted datasets, as well as transformation scripts, need to be made available.
*Response: We apologize for the issue with data accessibility. Our figshare database was still private. It has now been published and the doi link should now be accessible.*

- The SRA accession numbers must be listed for the sequencing datasets.
*Response: This information has now been included in ‘Data Availability’ section lines 634-635; SRA Bioproject number is PRJNA1416438.*

- The whole genome sequencing for variants is not sufficiently described. What was the method for classifying variants? It is unclear what is meant by "Due to variable sequencing coverage across the genome, mutations were only identified if they were found in each of a sample's sequences from the three primer set amplifications that had sequencing read coverage at that genetic locus." How is lack of variation differentiated from lack of sequence coverage? The authors should consider making the analysis a more significant part of the main text, and potentially include Supplementary Data Table 7 as a main figure with more text to address the above questions.
*Response: We thank the reviewer for this comment and have modified the text to increase the description of the analysis (lines 181-186), added details to the methods (lines 572-577), and moved supplemental table 7 into the main text as Table 1. Additionally, we have included a supporting figure (S3 Figure) to further describe the sequencing coverage for each of the samples.*

Minor comments
- The abstract is vague for findings that seem important to clarify. It notes that "Virus was detected in the air in milking parlors and from exhaled breath of cows" but does not clarify that for exhaled breath it was viral RNA, but that for milking parlors it was both viral RNA and infectious virus. It also notes that "sequence analysis revealed viral variants on a farm in these locations" but not that these variants seem relevant for potential human susceptibility. It further notes that "a heterogeneous distribution of infected quarters that maintained a consistent pattern over time" but not that the pattern is inconsistent with transmission due to shared milking equipment, thereby suggesting other modes of transmission may be occurring. These points seem like some of the main takeaways from the manuscript and it would benefit the clarity to directly explain them in the abstract.
*Response: We thank the reviewer for these points and have modified the abstract as suggested. Please see lines 39-40, 42, 45-46.*

- It is not clear what the motivation is for performing exhaled breath sampling from cows. Is it known whether virus-laden aerosols are generated from the cow respiratory tract? And if not, was this the motivation?
*Response: Several bovine diseases are presumed to transmit via aerosols, but we are not aware of any studies of virus-laden aerosols generated from the cow respiratory tract (PMID: 40872728). The initial surveillance studies observed sporadic nasal swabs positive for H5N1 (PMID: 39658318). While the viral loads from these swabs were low (high CT values), we were intrigued whether this implied that virus was replicating at low levels in the respiratory tract and was being released through the air. Therefore, exhaled breath was included in our sampling efforts, but is not currently thought to be the predominant mode of transmission.*

- The first sentence of the results section reads "During the winter of 2024". If this date range were clarified to include the months or even days it would help provide more context for the reader.
*Response: We thank the reviewer for the suggestion-- the line has now been clarified to include the month range, please see line 105.*

- Figure 1E shows superior performance of the MD8 Airport over the AirPrep Cub, but there is no elaboration on why this would be the case which is especially important given that the figure also demonstrates that the Cub is operating at a higher flow rate. Why would this result have occurred? Is it because of the mechanism of capture, because of the assay, or some other reason? Should these results even be compared, and were the methods of sampling the same when using both instruments (in the text it described exhaled breath sampling for the MD8 but not the Cub)?
*Response: We have clarified this observation and included a discussion of the possible reason for the discrepancy in the text, lines 126-129.*

- Air sampling is described in milking parlors and housing pens, but as a reader it is difficult to understand the nature of these environments. To what extent are they indoors/outdoors? How much outside air or sunlight do they receive? Could some of this contribute to dilution or degradation of viruses that might otherwise be expected?
*Response: We have added details to the legend in figure 1 to provide more information about the indoor vs outdoor air quality of these spaces. In addition, we have added text to the discussion for this point, please see lines 276-278. The following text was added to figure 1 legend: “While the specific layout and structures could vary by farm, the housing pens were typically open-air structures with only a roof, allowing continuous exposure to sunlight and outside air. Holding pens were largely within the indoor dairy parlor structure, with some outdoor air penetrating into this space. The dairy parlor is predominantly an indoor space with airflow occurring through open windows with or without screens.”*

- There is detection of live IAV in wastewater which is not necessarily normal for other wastewater studies. Can the authors comment on why this could be the case? What level of dilution could be happening that explains why we either should or shouldn't expect live viruses?
*Response: The wastewater sampled on dairy farms is distinct from human wastewater systems. In human wastewater systems, while viral RNA may be readily detected, infectious virus is less likely to be found due to the digestive processing that occurs when respiratory secretions are swallowed. On dairy farms, the waste milk may contain high titers of infectious virus, and we have previously shown that milk may stabilize H5N1 (PMID* *38914418). These factors increase the likelihood of finding infectious virus in wastewater on farms. However, as we state in the discussion, the low number of infectious samples we found in our study could have been due to other issues with sample fragility or processing issues. Thus, the detection of live H5N1 in waste streams on farms may be due to both less dilution at the various sites (higher infectious titers to start with), and potential stabilization of virus in the milk. We have adjusted the discussion accordingly, see lines 332-335.*

- For whole genome sequencing, there is missing information which would be useful to interpret the results. What was the depth and coverage of sequencing?
*Response: The depth of coverage was highly variable between samples/primers sets and across the genome, with many areas of missing coverage from some samples. However, a supporting figure (S3 Figure) with the individual plots per sample has now been included in the manuscript for full transparency.*

- Supplemental figure 3 shows the "Presence of anti-H5 HA antibodies in expressed milk from cows with subclinical symptoms". This seems like a major finding of the paper, fitting as a main figure rather than a supplementary figure.

*Response: We thank the reviewers for this comment and have included this figure in the main text – it is now Figure 4.*

- The text mentions "extended" tables multiple times. If this refers to supplementary tables then these should be listed as "supplementary tables".

*Response: We thank the reviewer for pointing out these inconsistencies and have corrected them for consistency.*

**Reviewer #2:**
In a comprehensive, data-driven study, the authors concluded that, in addition to virus-positive raw milk, there must be other potential transmission routes for HPAIV H5N1 in US dairy herds. To this end, they carried out detailed analyses of the air and wastewater in affected herds. Individual animals were also examined. It was found that even animals that appeared clinically sound seroconverted, and that animals that were virus-positive in milk did not necessarily develop mastitis. In animals affected by clinical mastitis, not all udder quarters were necessarily affected.

The authors derived meaningful mitigation strategies from these findings: Milkers should wear protective clothing that prevents exposure to aerosols, such as goggles and a mask. Milking equipment should be disinfected after each cow. Milk from infected animals should be disinfected before being passed on to the wastewater system. Subclinically infected animals should be identified using a simple, rapid test that can be carried out on the farm.

To date, experimental studies have shown that direct inoculation of the virus into the udder is necessary for infection. Infection via other routes (intranasal or intramuscular) did not lead to udder infection, and other organ systems are also spared from manifest infection. In this respect, it remains difficult to imagine how udder infections can be established through these alternative infection routes. The authors already envisaged contacts of (injured) teats with virus-positive waste water. Such options should be explored further in their otherwise highly interesting manuscript.
*Response: We thank the reviewer for recognizing the importance of our findings and their suggestion. We have added text to the discussion, please see lines 300-304.*

**Reviewer #3:**
This study from Campbell and colleagues describes surveillance conducted on California dairy farms with H5N1 detections during October, 2024-March, 2025. Both viral RNA and (in a subset of samples) infectious virus were sampled in a range of environmental locations, including the air of housing and milking pens, the exhaled breath of cows, various locations in the wastewater stream of infected farms, as well as longitudinal samples of milk from individual quarters of infected cows. The study detects the presence of viral RNA in the air of milking parlors on farms with confirmed H5N1 outbreaks (determined by the detection of viral RNA in bulk milk tanks), and in multiple reclaimed wastewater sites. The study also shows the presence of anti-H5 antibodies in the milk of dairy cows that were displaying subclinical symptoms at the time of sampling. Finally, the study shows that, in a subset of 9 longitudinally sampled cows, many cows are not positive in all four teats, suggesting
(as others have experimentally reported) that infection does not automatically spread from quadrant to quadrant in an infected cow. The question of how transmission of H5N1 influenza A virus is occurring amongst dairy cattle is of high significance for the field and has critical public health implications. The data here suggest that there is abundant viral RNA in many locations on infected dairy farms (particularly in milking parlors, and in wastewater streams). This is not necessarily surprising, as high levels of viral titers have previously been detected in milk of infected animals, and milk from animals known to be positive is generally discarded in the farm wastewater stream rather than being sent to the commercial milk supply. However, the title and conclusions of the manuscript go significantly beyond the data presented in this study, which does not answer the question of how transmission is occurring. Showing that viral RNA is in the air does not prove that
transmission occurs through this route. In addition, there are concerns about the lack of negative controls, the fact that methods to prevent pre-PCR contamination are not described, and differences in interpretation regarding whether viral RNA detected in the air is coming from splashes of milk or is being exhaled by cows. 
*Response: Our data has revealed the presence of infectious virus (not just viral RNA) in multiple locations on a dairy farm, highlighting other possible sources of transmission. We have adjusted the language in the title to include the word ‘possible’ to highlight that we do not conclusively establish a specific mode of transmission on dairy farms. We also have adjusted language throughout the manuscript to 1) temper conclusions drawn from RNA levels; 2) highlight whether the virus detection was for infectious virus by PFU or TCID_50_ ; and 3) to add discussion on all the possible modes of transmission. Technical concerns are addressed below.*

Major Points
*       The study (title and conclusions) appears to be drawing a link from viral RNA in the air, concluding this comes from viral RNA exhaled by cows, and then extending that to airborne viral transmission. However, H5N1 in the air in milking parlors as a side effect of the milking process is quite different from H5N1 in the air being breathed out by cows. Tt seems clear from this data that viral RNA can be found readily in the air in milking parlors (where there is abundant splashing of highly infectious milk, a scenario that seems likely to produce aerosols). However, the data for viral RNA in the air outside of this scenario seems considerably weaker. A Ct of 40 for RNA in exhaled breath (Fig 1F) or 4-40 genome copies (Fig 2C) is really borderline positive, and raises concerns about pre-PCR contamination.

*Response: We respectfully disagree with the reviewer’s assumption that the title and conclusions appear to link viral RNA in the air to only the exhaled breath of cows and airborne transmission as we do not make such conclusions. The text already highlights the potential to produce virus containing aerosols in the milking parlor from splashing and indicates that this is a source of infectious virus. In examining potential sources of transmission, it was critical to study the air in multiple locations, including from the breath of cows even though this may not be a major source of virus production. We also respectfully disagree with the reviewer’s comment regarding Fig 2C – the genome copies are per liter of air and these are not ‘borderline positive’ since we were capturing 50 liters of air per minute. However, for clarity and to be fully transparent with our data, we have adjusted the language in the title (added the word ‘possible’) and added in the qualifier ‘weakly positive’ for the data from exhaled breath present in Figures 1F and 2C. Please see lines 125 and 147-148.*

*       No precautions to avoid pre-PCR contamination are described. This is a significant concern, as pre-PCR contamination (from plasmid or viral RNA present in the lab) is a major concern and the difficulty of avoiding and eradicating such contamination  was extensively described during the SARS-CoV-2/COVID-19 response. No-template controls should be included on every plate, but a physically separate location from any amplified product/plasmid/viral RNA extractions is likely necessary to prevent contamination. Many of the conclusions described here are based on very high Ct or very low copies of genome, which is particularly worrying for the possibility of contamination. This is especially true with the modification of increasing the cycling from 40 to 45 cycles, which is an unusual change given the possibility of artifacts when extending over 40 cycles.

*Response: We thank the reviewer for this comment and have added additional information to the Materials and Methods section to highlight the rigor with which the RNA processing was undertaken at the Emory lab to ensure that the results were reproducible and accurate. This includes: 1) Generating aliquots for RNA extraction when the daily samples were received into the BSL3 lab at Emory such that they were not removed while other studies were being processed; 2) Addition of the lysis buffer (to the pre-aliquoted samples) in BSL3 and then subsequent RNA extraction in the BSL2 lab (per approved biosafety protocols); 3) RNA extraction via robot with inclusion of a negative extraction control (PBS) in each extraction run; 4) Positive viral detection required both sample replicates to each exhibit amplification (have CT values). We never observed any amplification (Ct values) in any of the negative extraction control (PBS) or the no template PCR control (water) wells from over 10 extraction plates and RT-qPCR plates run in this study; 5) Positives with a CT value >38 CT were reextracted and run again to confirm positivity; 6) All plates included serial dilutions of an in vitro transcribed H5 HA RNA standard with a known concentration for determination of genome copies. Please see the modified text on lines 474-497:*

*       The quantification of H5N1 viral RNA levels is crucial to the conclusions of this manuscript, but it is difficult to understand how samples can be directly compared as two different protocols (RNA extraction, RT-PCR reagents and platform, primers) were used throughout. In addition, some samples were detected via the use of a Swine Influenza Virus RNA Test Kit, which presumably relies on a universal IAV primer (since it appears to detect H5, which is not the predominant subtype in pigs). It is possible that the small number of positive (exhaled) air samples could be a non-H5 subtype (such as H1N1 or H3N2 released from a farm worker infected with a seasonal human subtype). Samples should be run with a second independent, H5-specific primer set, and negative controls should be included in the final figures to rule out environmental contamination (ie, from plasmid or viral RNA present in the laboratory environment).

*Response: We thank the reviewer for this comment. Field surveillance includes many challenges and early on in our study samples were processed close to the field site at Lander Veterinary Clinic (data presented in Figure 1), which could not assess the infectivity of any positive samples. Thus, the decision was made to shift analysis to Emory to allow for analysis of infectious titers in the various sample collections within our approved BSL3 space. It should be noted that the analysis done at Emory did use H5 HA specific primers as opposed to the Swine Influenza Virus RNA test kit used at LVC (please see updated Materials and Methods). Given that the techniques are slightly different, we avoid making comparisons across the two sampling efforts. However, since we observe consistency across those sampling efforts (the presence of H5N1 in the air within dairy parlors and weakly positive samples from exhaled breath of clinically sick cows), we feel that the use of slightly different methods increases the confidence in conclusions and argues against a protocol-specific issue responsible for the results. Details have been added to the Materials and Methods as well as to the figure legends to expand on the process of RNA quantification and control methods undertaken at Emory for the bulk of sample processing. Please see revised Figure 2 legend, and lines 474-497 of the Methods section.*

*       As both plaque assays and TCID50s are used (sometimes in the same figure), pictures of the raw assays would help considerably in interpretation. This is especially true for samples in which the plaque titer indicates that a very small number of plaques were present (Fig 2D, Fig S2B). In Fig 3C, it should be clarified whether PFU or TCID50 is being used for each data point. For TCID50, it should be clarified whether other causes of CPE can be ruled out (as bacterial contamination would also cause cell death, and it is likely there is a high level of bacteria in the manure lagoon sample in particular).

*Response: For the samples in Figure 3C, some were assessed by PFU, others by TCID50, and some by both assays, with the highest titers presented. We have now modified Figure 3C to indicate which assay was used for each result. We have also clarified in the Materials and Methods that TCID50 assays were performed with double the concentration of antibiotics and antimycotics, and any wells with bacterial contamination were verified to be virus positive by either HAI titer of the cell supernatant or by serial passage of a small volume onto another plate of cells with antibiotics and antimycotics to ensure that the CPE was driven by virus. Standard practice is to also use crystal violet staining of the TCID50 monolayer to ensure that there are holes in the monolayer. Please see lines: 522-534.*

*       Just because contaminated milking equipment does not appear to be the sole determinant of transmission does not mean that alternative mechanisms of cow-to-cow transmission must exist. Transmission could occur entirely via contaminated equipment, but ALSO require a particular set of intrinsic host/cellular determinants (cellular state, local innate immune state, quadrant specific tissue damage, etc). It seems much more likely that animals with damage to their teats are infected through contact with contaminated milking equipment, vs their teats contacting aerosols or wastewater.

*Response: Currently, it is not clear what host factors are involved, and the lack of individual cattle surveillance has hindered our ability to define transmission rates. We have extended the discussion in the manuscript to include alternative transmission routes and discuss the possibilities suggested above (see lines 300-304). While it is likely that host factors are also at play for susceptibility and forward transmission of H5N1 on dairy farms, ignoring the spaces on a farm that contain infectious H5N1 and other possible modes of transmission will prevent containment of outbreaks.*

*       The point that "the lack of a consistent quarter infected across all cows, as observed for mastitis, suggests that these two features are not linked and likely have distinct modes of transmission" seems to be overstating the case. While 6 of 9 cows (from a single farm) had mastitis in the FR quadrant, and the presence of H5 RNA was more equally distributed among quadrants, it could be possible that H5N1 infection is a pre-requisite for mastitis development. The apparent over representation of mastitis in the FR quadrant could also have a mechanical explanation, as frequent and efficient removal of milk is needed to prevent mastitis symptoms from developing. If the suction or efficiency of the milking equipment that was repeatedly used for the FR quadrant was somewhat less than the other three quadrants, that could explain the preponderance of mastitis there.

*Response: We have added this is possible caveat in the discussion. Please see lines 314-317.*

*       The title and general conclusions greatly overstate what can be concluded by the data. The authors have found some evidence that H5N1 RNA can be found in multiple places within dairy farms with infected herds. This does not prove in any way how transmission is happening, and certainly not that it is happening through multiple different routes.

*Response: We have tempered conclusions and the title of the paper as suggested, but maintain that our data highlight that other H5N1 sources and modes could be playing a role in spreading H5N1 on a farm.*

Minor Points
*       As this study relies on procedures involving animals it seems there should be an IACUC review/protocol associated with it.

*Response: We have included an animal ethics statement in the Materials and Methods that states that our sampling efforts are IACUC exempt (lines 378-384).*

*       Hard to compare Ct to genome copies, a standard curve comparison should be included.

*Response: We thank the reviewer for highlighting this. We have now mentioned that an in vitro transcribed H5 RNA standard curve was added to each qPCR plate for determination of Ct to genome copies. A representative standard curve is provided here for this reviewer.*

*       The sequencing data seems as if it could be very valuable, but is underexplored. It is also unclear which mutations (in supplemental data table 7) are detected in primary samples, vs in cell passaged stocks. The methods section indicates that five of the sequenced air samples include a cell passaged stock, but it is unclear which of the samples in Table S7 this corresponds to. There is also reference to mutations that appear but are not maintained, but it is unclear how this conclusion is supported. Are the same cows sampled over time, so individual mutations can be seen appearing and disappearing? Even in this scenario, with such low genome copies captured, it seems entirely likely that mutations could simply not have been captured upon resampling.

*Response: We thank the reviewer for this response and have moved the table to the main text (now Table 1). Additionally, we have updated the table to indicate which samples are cell propagated and which ones are not. We are currently analyzing the variation of viral variants within a cow over time and this will be a separate analysis. Thus, we have deleted the reference to variants not being maintained as this will be clearer from repeated individual cow sampling as stated by the reviewer.*

**Reviewer #4:**
Authors conducted individual animal sampling (milk from all quarters, nasal swabs) without ethics approval. This reviewer believes that IACUC approval should have been obtained prior to animal sampling and would like that concern to be addressed before proceeding with full manuscript review.

*Response: As mentioned in the response to reviewer #3, we have included an animal ethics statement in the Materials and Methods (lines 378-384) that states that our sampling efforts are IACUC exempt based on determination from our institutional review committees.*
